# Study on the Mechanism of Lianpu Drink for the Treatment of Chronic Gastritis Based on Network Pharmacology

**DOI:** 10.1155/2021/6693472

**Published:** 2021-04-17

**Authors:** Shuhan Zhou, Yanjun Duan, Yu Deng, Miao Wang, Chaoqun Huang, Zhiyi Liu, Xiaohui Xu, Wenliang Lv

**Affiliations:** ^1^Clinical College, Hubei University of Chinese Medicine, Wuhan 430000, China; ^2^College of Basic Medicine, Hubei University of Chinese Medicine, Wuhan 430000, China

## Abstract

Chronic gastritis (CG) places a considerable burden on the healthcare system worldwide. Traditional Chinese Medicine (TCM) formulas characterized by multicompounds and multitargets have been acknowledged with striking effects in the treatment of CG in China's history. Nevertheless, their accurate mechanisms of action are still ambiguous. In this study, we analyzed the effective compounds, potential targets, and related biological pathway of Lianpu Drink (LPD), a TCM formula which has been reported to have a therapeutic effect on CG, by contrasting a “compound-target-disease” network. According to the results, 92 compounds and 5762 putative targets of LPD were screened; among them, 8 compounds derived from different herbs in LPD and 30 common targets related to LPD and CG were selected as candidate compounds and precision targets, respectively. Meanwhile, the predicted common targets were verified by Kyoto Encyclopedia of Genes and Genomes (KEGG) signaling pathway analysis and pharmacological experiments. The results demonstrated that quercetin, ephedrine, trigonelline, crocetin, and *β*-sitosterol were major effective compounds of LPD responsible for the CG treatment by inhibiting the activation of the JAK 2-STAT 3 signaling pathway to reduce the expressions of cyclin D1 and Bcl-2 proteins. The study provides evidence for the mechanism of understanding of LPD for the treatment of CG.

## 1. Introduction

Chronic gastritis (CG) is a common disease of atrophy of the gastric glands and mucosal epithelium caused by the loss of the gastric mucosal epithelium with specific regenerative ability after being stimulated [1]. Although gastritis plays an important role in the pathogenesis of common peptic ulcer and gastric cancer, the importance of CG as a serious disease has been underestimated to a large extent in clinical practice [2–5]. If it is not treated in a timely and effective manner, CG can easily develop into gastric cancer [6, 7]. Gastric cancer is the second most common cancer worldwide after lung cancer. It is estimated that millions of people worldwide may die from cancer and ulcers each year as a consequence of chronic gastritis [8].

Through the continuous research, development, and innovation of TCM scientists and pharmacists, Chinese medicine has become the national medicine for treating various diseases in China [9]. As far as CG is concerned, many Chinese herbal medicines and TCM formulas have been successfully used to treat CG. In TCM science, according to the etiology and pathogenesis of CG, it is mainly divided into five types, namely, liver-stomach discord, stomach-yin deficiency, spleen and stomach damp heat, blood stasis stagnation, and spleen-gastric weakness [10]. It is very important to choose appropriate drugs for the treatment according to the different types of CG. TCM formulas characterized by multicompounds and multitargets have been acknowledged with striking effects in the treatment of CG in China's history. Clinical trials and statistical analysis demonstrated that Chaihu-Shugan-San can promote gastrointestinal tract activity, eliminate stasis, and accelerate the repair of the gastric mucosa in CG patients caused by liver-stomach discord [11]. The TCM compound such as Dendrobium Yangwei decoction can achieve the therapeutic effect on CG induced by deficiency of stomach yin through nourishing the yin and stomach and moderate pain relief [12]. In clinical practice, Huoxue Zhitong decoction is usually used to treat blood stasis and gastrointestinal CG by regulating Qi and activating blood circulation, dredging collaterals, and relieving pain [13]. Moreover, clinical studies showed that Banxia Xiexin decoction, Sanren decoction, and LPD have an obvious effect on the treatment of CG patients induced by spleen and stomach damp heat [14–16]. Nevertheless, their accurate mechanisms of action are still ambiguous.

Uncovering the association between Lianpu Drink (LPD) and CG was the focus of this study. The original prescription of LPD is from the “*Cholera Theory*” written by Wang in the Qing Dynasty. It was originally used to treat damp and hot cholera. The composition of LPD was Coptidis Rhizoma (CR), *Magnolia officinalis Rehd Et Wils*. (MOR), *Cape jasmine* (CJ), *Pinellia ternata* (PT), *Acorus tatarinowii* (AT), fermented soya beans (FSB), and reed rhizome (RR), which is a classic prescription for CG. The characteristics of CR and CJ are bitter taste and cold nature, and they have the effects of clearing heat, purging fire, and drying dampness. CR and CJ have been proved to have strong anti-inflammatory activities [17, 18]. Meanwhile, MOR, PT, and AT are compatible with each other in LPD, combined with bitter taste and pungent smell, and have the functions of drying dampness and turbidity. PT has the function of harmonizing the stomach and reducing the adverse Qi. A previous study demonstrated MOR involved Houpu Sijunzi decoction has a significant clinical effect in the treatment of CG [19]. Besides, it has been reported that PT plays a role in protecting the gastric mucosa and promoting the repair of it by reducing gastric juice secretion, reducing free and total acidity of gastric juice, and inhibiting pepsin activity [20]. Meanwhile, AT is one of the important components of stomach evacuation prescription. Moreover, FSB can release depression and heat. Also, RR has the effect of clearing heat and promoting fluid. The combination of these various medicines is a good prescription for drying dampness and clearing heat [21, 22].

TCM is a complex system of multifarious components and targets, which has the characteristics of synergistic effects of various compounds in its function in the body [23]. Although many TCM products have achieved good clinical efficacy in the treatment of CG and other diseases, the mechanism of their efficacy *in vivo* is not clear. Especially, we still face many problems in the research of the mechanism of the compound medicines. The traditional theory of “single compound”-“single target” cannot satisfy the research of “multicompound”-“multitarget”-“multipathway” interaction of TCM [24]. However, network pharmacology (NP) is a system of biology and multidirectional pharmacology combination, emphasizing that the role of drugs is to intervene in disease networks. Its concept is consistent with the characteristics of TCM compound. It provides a new strategy for the mechanism research of TCM compound. NP is a new strategy and method for the research and development of new drugs, which is based on the theoretical development of systems biology, multidirectional pharmacology, histology, and other disciplines. The “drug-target-disease” network is established by analyzing the existing information including genome, proteome, drugs, diseases, and other related databases and then combining with the experimental verification data. Then, the computer network analysis software is used to display the relationship between drugs and targets, between targets and diseases, between diseases and diseases, and between drugs and drugs. From this, we can observe the intervention and influence of drugs on diseases from the network level and reveal the mechanism of drugs in the human body, so as to find out low-toxicity and high-efficiency multitarget drugs [25, 26].

In this study, we constructed a database of all the molecules contained in the 7 herbs in LPD and selected oral bioavailability (OB) and drug-like properties (DL) as screening parameters to obtain potential pharmacodynamic compounds based on the pharmacological method of the Chinese medicine system. The GeneCards database was used to predict the proteins that can target these pharmacological compounds. Moreover, we analyzed the effective compounds, potential targets, and related biological pathways of LPD by contrasting a “compound-target-disease” network. Based on this, we explored the mechanism of LPD in treating CG. Finally, in order to prove the accuracy of the results obtained by computer algorithms and prediction models, we verified the effects of several candidate compounds through pharmacological experiments.

## 2. Materials and Methods

### 2.1. Prediction of the Active Components and Target Proteins of LPD Related to CG by NP

#### 2.1.1. Collection of the Candidate Compounds of LPD and Prediction of Its Targets

The compounds of CR, MOR, CJ, PT, AT, FSB, and RR were collected by TCMSP (http://tcmspw.com/tcmsp.php) and BATMAN-TCM (http://bionet.ncpsb.org/batman-tcm) databases. In order to improve the accuracy of compound prediction results, the compounds were screened with oral bioavailability (30% or more), drug-like properties (0.18 or more), or score cutoff (20% or more) as evaluation indicators. OB is a good indicator of the efficiency of oral administration for drug delivery into systemic circulation [23]. DL is a qualitative property of chemicals that describes the pharmacokinetic and pharmaceutical properties of compounds, which can be used to evaluate whether a compound is drug-like or not [27]. The ingredients that satisfied both OB ≥ 30% and DL ≥ 0.18 thresholds were regarded as candidate compounds in the TCMSP database. Meanwhile, for each compositive compound of LPD in the BATMAN-TCM database, the putative targets whose scores given by the target prediction method exceed a given cutoff “score cutoff ” (≥20%) will be considered as the potential targets. Moreover, the target proteins of the screened compounds were imported into the STRING 10.5 database (https://string-db.org). Then, they are corrected to their official names, and their standard gene names are extracted and will be presented and further analyzed.

#### 2.1.2. Prediction of the Targets of LPD on CG

GeneCards is a searchable integrated database that provides some important information for us to understand the relationship between disease target proteins and human disease. Our study was focused on the effect of LPD on treating CG. So, “chronic gastritis” as a keyword related to disease helped us find potential target in the GeneCards database. With “Chronic Gastritis” as the key word, the relevant targets of CG were searched by the GeneCard database (https://www.genecards.org). Targets with a correlation score of 20 or more were considered as precision targets for CG. The common targets of compounds and CG were obtained by importing the target proteins of drugs and disease into the Venny 2.1 database (https://bioinfogp.cnb.csic.es/tools/venny/index.html). Furthermore, a “compound-target” map was made using Cytoscape software.

#### 2.1.3. Construction of the Protein-Protein Interaction (PPI) Network between LPD and CG Targets

We upload the common targets obtained in here to the STRING10.5 database (https://string-db.org), select the research species as “humans,” and set the minimum connection score between the targets as 0.4. The map of the target connection network diagram was drawn and then introduced into Cytoscape software to build the PPI network. The CG target's PPI network was constructed, and the noteworthy features of the network analyzed could provide some important information for us to understand the “target-target” interaction mechanism.

#### 2.1.4. KEGG Pathway Analysis

The KEGG pathway, a knowledge base including most of the known metabolic pathways and some of the known regulatory pathways, can provide important information for us to uncover the mechanism of a certain drug on disease [28, 29]. KEGG pathway analysis was performed to predict the molecular interactions and reaction networks associated with differently regulated genes. The common targets of LPD composition and CG were imported into the DAVID database (https://david.ncifcrf.gov), and then, “KEGG” was selected for annotation analysis of the target gene pathway. The results of KEGG analysis (meeting *p* < 0.05) were made into a visual bubble and cluster chart by omicshare and Heml software, respectively. Moreover, the most significant pathway was screened out to verify the mechanism of LPD in treating CG by analyzing the key target proteins and their downstream proteins in the pathway.

### 2.2. Experimental Validation

#### 2.2.1. Preparation of LPD

According to the original formula ratio, weighed 12 g of MOR, 6 g of CR, AT, and PT, 18 g of FSB and CJ, and 120 g of RR, respectively, were added to 200 mL of water and soaked for 30 min. Then, 200 mL of water was added, decocted for 15 min, filtered, and concentrated to 186 mL. LPD with a concentration of 1 g/ml was obtained and put in the refrigerator at 4°C for standby.

#### 2.2.2. Animal Modeling and Grouping

290 Sprague Dawley (SD) male rats (100–130 g, 3–4 weeks) were purchased from Beijing Weitong Lihua Experimental Animal Technology Co., Ltd., China (SCXK(Jing)2018–0010) and kept in a standard environment in the lab animal room in the clinical college, Hubei University of Chinese Medicine. The CG model in rat was established according to the methods described by previous studies [30, 31]. In brief, the CG rat model was induced by a comprehensive method based on *N*-methyl-*N*′-nitro-*N*-ni-trosoguanidine (MNNG, concentration 150 *μ*g/mL) free drinking, changed daily. Moreover, daily free eating including 0.05% ranitidine granular SPF-grade rat feed, there was no other food during the period, was accompanied by the way of hunger and satiety disorder, that is, even-numbered days of full food and odd-numbered days of fasting. During one-day fasting, rats were administered 2% sodium salicylate solution 0.5 mL/100g. The control group were given normal feed and drinking water. Model establishment lasted 10 weeks.

Rats were randomly divided into 29 groups (*n* = 10), namely, the control group, model group, quercetin low-, middle- and high-dose groups (quercetin-L, quercetin-M, and quercetin-H), ephedrine-L, ephedrine-M, ephedrine-H, jasmone-L, jasmone-M, jasmone-H, trigonelline-L, trigonelline-M, trigonelline-H, crocetin-L, crocetin-M, crocetin-H, *β*-sitosterol-L, *β*-sitosterol-M, *β*-sitosterol-H, *β*-asarone-L, *β*-asarone-M, *β*-asarone-H, valine-L, valine-M, valine-H, LPD-L, LPD-M, and LPD-H, respectively. After modeling, rats in the control group were administrated with 0.9% normal saline, whereas other groups were administrated with hypodermic injection with different potential active compounds (7.5 mg/kg, 15 mg/kg, and 30 mg/kg) or LPD (0.50 ml/100 g, 1.0 ml/100 g, and 1.5 ml/100 g) everyday, lasting for 10 weeks, respectively.

#### 2.2.3. ELISA Assay

5 mL of arterial blood was extracted from the femoral artery of rats, and the serum was separated and stored at −20°C. Pepsinogen I (PG I), pepsinogen II (PG II), and gastrin-17 (G-17) ELISA kits were purchased from Shanghai Yuanye Bio-engineering Co., Ltd. (Shanghai, China). Moreover, p-JAK 2, p-STAT 3, cyclin D1, Bcl-2, TNF-*α*, IFNG, IL1B, IL6, and IL10 ELISA kits were purchased from Abcam (China). The levels of these factors were measured according to the operation instructions of the kits.

### 2.3. Statistical Analysis

Data are represented as mean ± SEM of independent experiments. Statistical analysis was performed using ANOVA and Student's *t*-test (two tailed). *p* < 0.05 was considered significant.

## 3. Results

### 3.1. Computational Prediction by NP Analyses

#### 3.1.1. Collection of Candidate Compounds of LPD

To give an accurate screening of the compounds of CR, MOR, CJ, PT, AT, FSB, and RR in LPD, an effective search method and filter conditions were conducted (the specific methods can be seen in Table 1). Based on the TCMSP and BATMEN databases [32, 33], the compounds of the seven single drugs including CR, MOR, CJ, PT, AT, FSB, and RR, as well as the putative targets corresponding to these compounds, were collected, respectively. Results showed that 92 compounds and 5762 putative targets were obtained with OB ≥ 30% and DL properties ≥0.18 or score cutoff ≥20%. Among them, 20 compounds and 420 of their putative targets belong to CR, 6 compounds and 194 of their putative targets belong to MOR, 14 compounds and 1129 of their putative targets belong to CJ, 25 compounds and 2696 of their putative targets belong to PT, 17 compounds and 1064 of their putative targets belong to AT, 3 compounds and 42 of their putative targets belong to FSB, and 7 compounds and 217 of their putative targets belong to RR. As shown in Table 1, in order to improve the screening accuracy of the abovementioned compounds, three important topological parameters of “betweeness centrality,” “closeness centrality,” and “degree” were used as evaluation indicators. We finally determined 8 compounds derived from different herbs in LPD and their target proteins as the research object.

#### 3.1.2. The Prediction Results of LPD on the Target of CG

**Figure 1:**
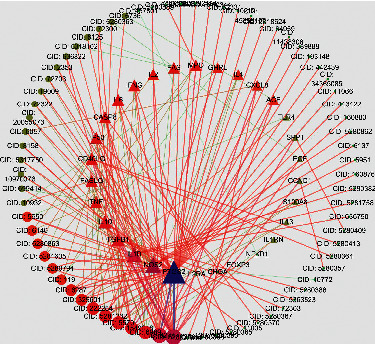
The “compound-target-disease” network for the treatment of CG with LPD. The triangles represent the targets of CG, and the dots represent the compounds of LPD.

A total of 958 targets related to CG were retrieved, and 52 targets with the correlation score ≥20 were selected as potential disease targets. Indisputably, herbal medicines exert their therapeutic effects through the synergy of effective compounds, compounds targets, and disease targets [34]. Therefore, the common targets for drugs and disease were important for further screening of key targets [29]. VENNY2.1 software, as a useful tool for finding common targets, successfully screened out 30 common targets related to LPD and CG. As shown in Table 2, the protein and gene names of each common target were listed according to the correlation score. In order to clearly explain the “drug-target” interaction mechanism of LPD compounds on CG, the “compounds-target-disease” network was constructed. As shown in Figure 1, the dots represent the compounds of LPD. The darker color and the larger area indicate that the compound has more CG targets. The triangles represented the target proteins of CG. The darker color and the larger area indicate that more compounds were involved. Moreover, a total of 255 pairs of LPD compounds and CG targets were chosen to screen the potential CG targets that LPD might act on according to the network analysis. Furthermore, the number of precision targets for each candidate compounds is shown in Table 1.

#### 3.1.3. PPI Network of Common Targets between CG and LPD

**Figure 2:**
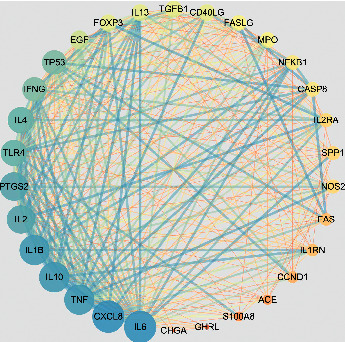
PPI network of common targets between CG and LPD.

In Figure 2, to clearly analyze the PPI network of the CG targets related to LPD, Cytoscape3.6.1 software as a visualization tool for complicated network analysis was performed, and the relationships between targets and targets were illustrated by different lines and dots. The dots represented the common targets. The darker color and the larger area suggest that this target has more connections with other targets. The lines represented the relationships between different targets; the darker color and the wider line evidence the closer connection for the different targets. Moreover, there were 294 connections between 30 common targets, with an average coordinate of 19.6 and a PPI enrichment value of <1.0*e* − 16 based on the network analysis.

#### 3.1.4. Pathway Analysis of LPD Treating CG

**Figure 3:**
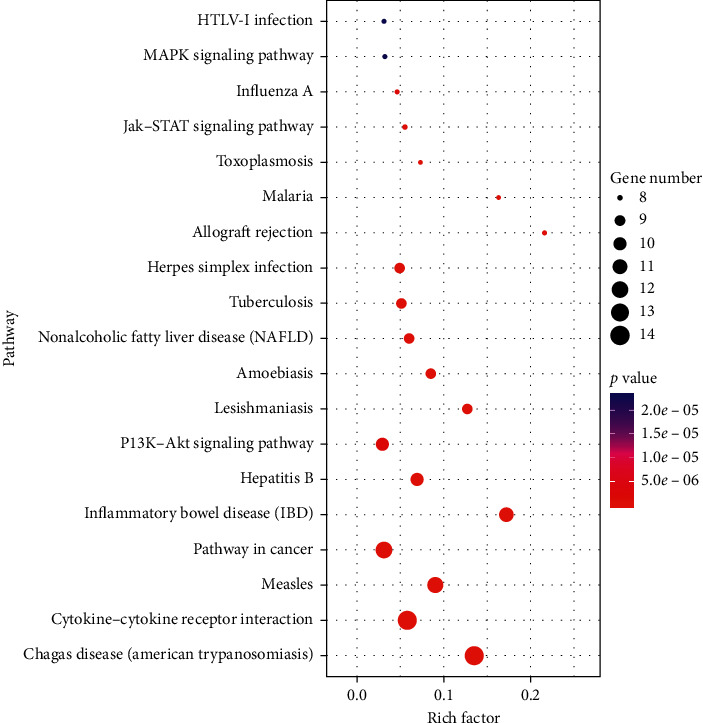
KEGG pathways analysis of LPD treating CG.

As shown in Figure 3, 19KEGG pathways with count ≥8 and *p* < 0.001 were enriched in the DAVID website. Through KEGG pathway analysis, it was found that LPD could treat CG mainly by interfering with the pathway in Chagas diseases, cytokine-cytokine receptor interaction, measles, cancer, inflammatory bowel diseases, and hepatitis B, P13K-Akt signaling pathway, Jak-STAT signaling pathway, MAPK signaling pathway, and so on.

### 3.2. Verification of the Effects of Candidate Compounds on CG

**Figure 4:**
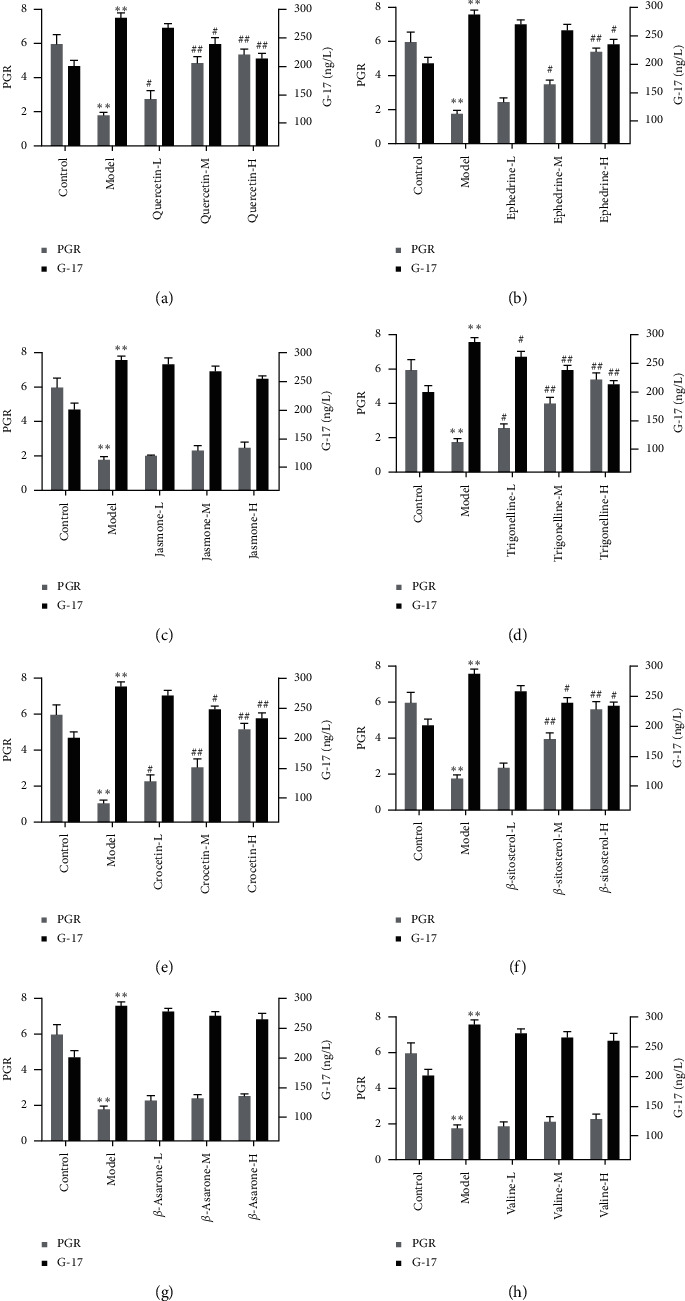
Effects of quercetin, ephedrine, jasmone, trigonelline, crocetin, *β*-sitosterol, *β*-asarone, and valine on CG rats. Model groups were compared with the control groups, ^*∗*^*p* < 0.05, ^*∗∗*^*p* < 0.01. Potential active compounds groups were compared with the model groups, ^#^*p* < 0.05, ^##^*p* < 0.01.

To further verify the effects of quercetin, ephedrine, jasmone, trigonelline, crocetin, *β*-sitosterol, *β*-asarone, and valine on CG, the levels of PG I, PG II, and G-17 in different groups were detected in this study. The results indicated that compared to the control group, the rate of PG I to PG II (PGR) was significantly decreased while the G-17 level was obviously increased in the model group (Figure 4). The results showed the CG model was successfully established. Furthermore, PGR was significantly improved while G-17 expression was remarkably decreased by quercetin, ephedrine, trigonelline, and crocetin, and *β*-sitosterolin in a dose-dependent manner compared with the model group, respectively. These results demonstrated that quercetin, ephedrine, trigonelline, crocetin, and *β*-sitosterol did have good effects on CG.

### 3.3. Effects of LPD on Jak-STAT-Signaling-Pathway-Related and Downstream Target Proteins

**Figure 5:**
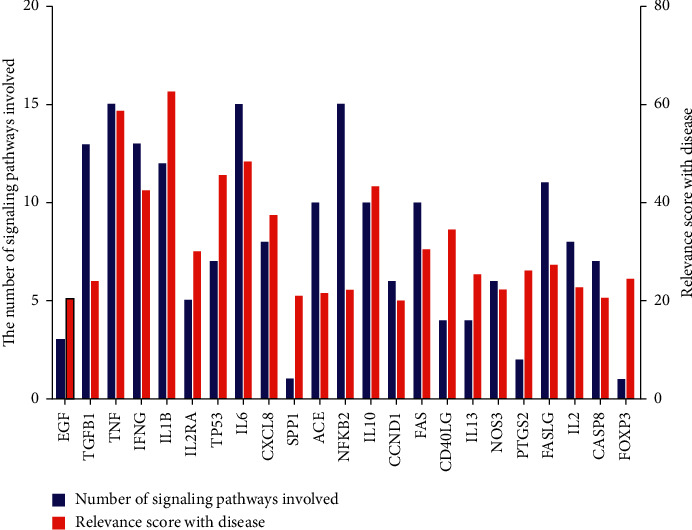
The collaborative study for common targets about the number of signaling pathways involved and the relevance score with disease.

Combined with the analysis results in Table 2 and Figures 3 and 5, we found that LPD and CG have 30 common target proteins. Among the many signal pathways related to these targets, 19 involved the most. A total of 23 nonredundant genes were annotated into these pathways. However, 16 genes appeared 5 times or more in these 19 pathways, including TGFB1, TNF, IFNG, IL1B, IL2RA, TP53, IL6, CXCL8, NFKB1, IL10, CCND1, FAS, NOS2, FASLG, IL2, and CASP8. Moreover, 6 of the 16 genes with a CG-disease-related score greater than 40 were TNF, IFNG, IL1B, TP53, IL6, and IL10.

**Figure 6:**
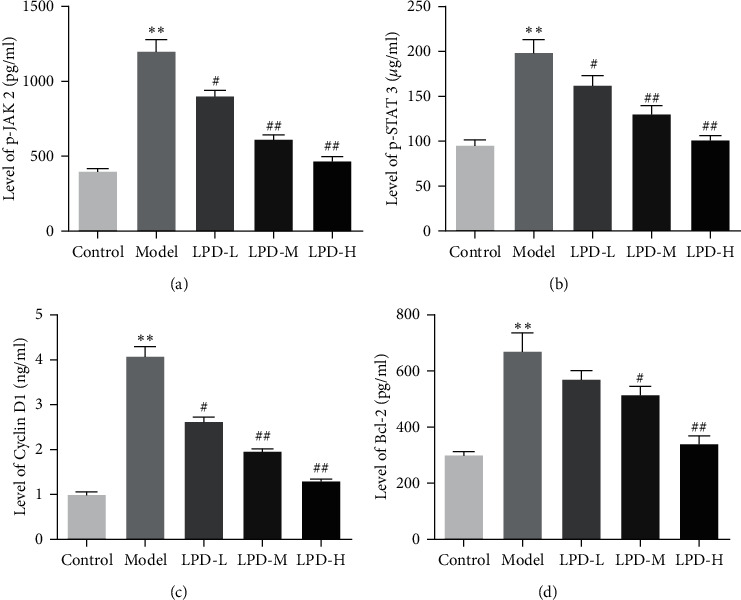
Effects of LPD on the Jak-STAT signaling pathway. Model groups were compared with the control groups, ^*∗*^*p* < 0.05, ^*∗∗*^*p* < 0.01. LPD groups were compared with the model groups, ^#^*p* < 0.05, ^##^*p* < 0.01.

**Figure 7:**
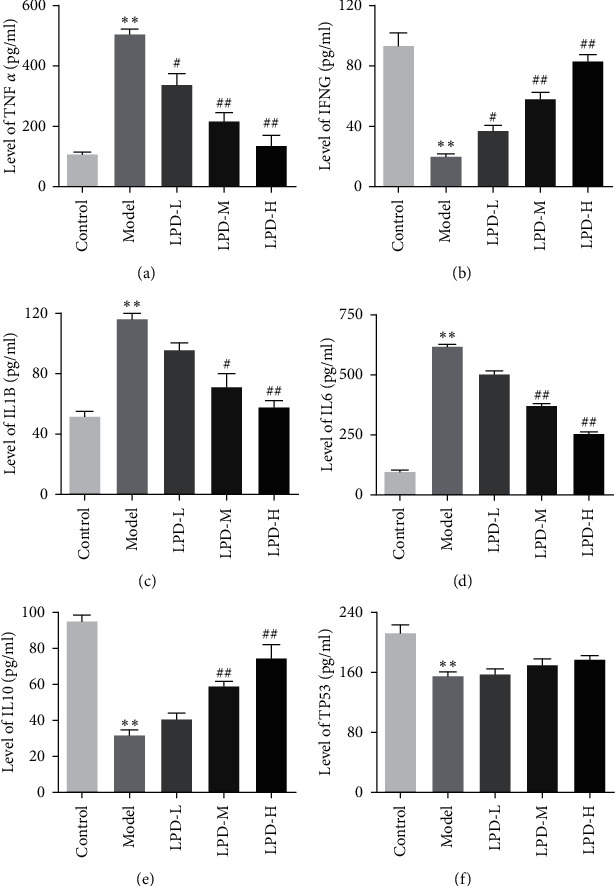
Effects of LPD on downstream targets. Model groups were compared with the control groups, ^*∗*^*p* < 0.05, ^*∗∗*^*p* < 0.01. LPD groups were compared with the model groups, ^#^*p* < 0.05, ^##^*p* < 0.01.

As shown in Figure 6, compared with control groups, the levels of p-JAK 2, p-STAT 3, cyclin D1, and Bcl-2 in model groups were significantly increased. Furthermore, compared with model groups, LPD remarkably decreased the levels of p-JAK 2, p-STAT 3, cyclin D1, and Bcl-2 in a dose-dependent manner. Meanwhile, in order to explore whether the inhibition of LPD on the JAK signaling pathway has an effect on downstream targets, we detected the expressions of TNF-*α*, IFNG, IL1B, TP53, IL6, and IL10. The results indicated that compared with control groups, the expressions of TNF-*α*, IL1B, and IL6 were significantly increased, while IFNG, IL10, and TP53 were obviously decreased in CG rats in a dose-dependent manner (Figure 7). Moreover, compared with model groups, LPD significantly reduced TNF-*α*, IL1B, and IL6 expressions, while remarkably improving the levels of IFNG and IL10 in a dose-dependent manner.

## 4. Discussion

LPD, as a classical prescription, has been used in clinical treatment on CG for thousands of years [35]. Liao et al. [36] reported that LPD can repair the damage of gastrointestinal mucosal cells and inhibit the inflammatory response of the body by regulating the balance between TH1 and TH2. Huang et al. [37] found that LPD can restore the antioxidant function of the body and reduce the heat source and inflammatory response by regulating the content of NO in the serum. However, the compounds and putative targets of LPD action against CG remain unclear. Therefore, further investigation is required.

In the research, a “compound-target-disease” network based on TCMSP and BATMEN databases was established. Effective compounds and target protein of LPD against CG were screened effectively. The predicted results indicated that 8 candidate compounds derived from different herbs in LPD have close connections with 30 targets of CG. Meanwhile, a compound can act on multiple target proteins, while a target can be acted on by multiple compounds. It suggested that LPDs exert their therapeutic effects through the synergistic effects of multiple compounds from different herbs and targets. In addition, the screened 30 common targets related to LPD and CG suggested that these targets might play an important role in the CG, and this further explains that the herbal medicine might act on the polypharmacological level, rather than on one specific protein, in order to combat complex diseases. The prediction results were consistent with the guess that the herbal medicines exert their therapeutic effects through the synergistic effects of multiple compounds and targets [38].

Although the predictive accuracy of network pharmacology has been verified by many studies [38, 39], the pharmacological verification experiments are necessary. In this study, we took the levels of PG I, PG II, and G-17 as an evaluation index of CG, and 8 candidate compounds effects acted on GC were verified. The results indicated that quercetin, ephedrine, trigonelline, crocetin, and *β*-sitosterol did have good effects on CG. Among them, quercetin belongs to CR, ephedrine, trigonelline, and *β*-sitosterol belong to PT, and crocetin belongs to CJ. The results demonstrated that LPD exerts therapeutic effects on GC through the synergistic effects of multiple compounds. A previous study showed that Quercetin is a perfect anti-inflammatory and antioxidant agent that has potential as an adjuvant treatment for inflammatory diseases and oxidative stress [40]. There is evidence which demonstrated that Ephedrine has a potent anti-inflammatory activity against D-GalN/LPS-induced acute liver failure in rats, and this comprehensive anti-inflammatory effect may result from the inhibition of TNF-*α* production [41]. Besides, trigonelline is a plant alkaloid and a major component of coffee and fenugreek with antidiabetic, antioxidant, anti-inflammatory, and neuroprotective effects [42]. Moreover, a large number of evidence showed that Crocetin and *β*-sitosterol have good anti-inflammatory effects [43, 44]. However, our study was the first one to report the effects of these five effective compounds on CG, although it was not thorough enough.

Moreover, our analysis results indicated that LPD may improve CG by interfering with multiple signaling pathways or biological processes. Among the many possible signal pathways, we selected the classic Jak-STATs signal pathway related to CG for verification. The Jak/STAT signaling pathway is one of the three known inflammatory signaling pathways, which plays an important role in digestive system inflammation [45]. A study indicated that various proteins related to the STAT pathway were abnormally expressed in CG patients [46]. At present, it is known that a variety of cytokines and growth factors are signal-transduced by activating the JAK/STAT pathway [47]. After binding cytokines and growth factors to their corresponding receptors, JAK on the surface of the cell membrane is activated to phosphorylate them, further activating the STAT in the cytoplasm, entering the nucleus and binding to specific DNA fragments, and regulating the expression of related genes, which involve cell proliferation, apoptosis and immune regulation, and other biological effects. STAT3 has been identified as an oncogene, which can mediate the transcription of various cytokines and growth factor signals to the nucleus, affect the transcription of target genes, and upregulate the proto-oncogene (c-Myc) by stimulating cell proliferation and inhibiting apoptosis [48]. Moreover, STAT3 can upregulate the role of genes such as C-Myc by stimulating cell proliferation and inhibiting decomposition participate in the formation of tumors [49, 50]. C-Myc plays an important role in controlling cell growth, differentiation, apoptosis, and tumor transformation and participates in the process of gastric mucosa intestinalization and canceration. Cytokine signal inhibitors can inhibit the cytokine-mediated JAK/STAT pathway, thereby putting the body in a dynamic balance, and then participate in the process of gastric mucosa intestinalization and canceration [51, 52]. Breaking the JAK/STAT signaling pathway may become a new way to treat tumors. In our study, LPD remarkably decreased the levels of p-JAK 2, p-STAT 3, cyclin D1, and Bcl-2 in CG rats. These results demonstrated that LPD may improve CG by inhibiting the activation of the JAK 2-STAT 3 signaling pathway to reduce the expressions of cyclin D1 and Bcl-2 proteins.

The results of this study showed that LPD can decrease the G-17 level and increase PGR in rats with CG. PG is a precursor of pepsin, which can be divided into two subspecies of PGI and PGII. With the progress of gastric mucosal inflammation, PGI in the blood gradually decreased, and the main cells were gradually replaced by pyloric gland cells. Meanwhile, PGII slightly increased or remained unchanged, and PGR also gradually decreased. Therefore, the serum PGI and PGII levels not only reflect the number of glands and cells in the gastric mucosa but also indirectly reflect the secretion function of different parts of the gastric mucosa. However, PGR can better reflect the degree of gastric atrophy than PGI and PGI [53]. The levels of PGI and PGR in CG are positively correlated with the degree of atrophy. Low levels of serum PGI and PGR can be used as a marker for screening high-risk groups of atrophic gastritis and gastric cancer. A previous study showed that PGI and PGR decreased in patients with atrophic gastritis [54]. With the appearance of intestinal metaplasia and moderate and severe dysplasia, they developed into precancerous lesions, and PGI and PGR also showed a downward trend. At the stage of gastric cancer, PGI and PGR levels will continue to decrease. Furthermore, G-17 is an endocrine hormone that regulates digestive tract function and maintains the integrity of the digestive tract. Its physiological functions include promoting gastric acid secretion, promoting gastric mucosal cell proliferation, and regulating gastrointestinal motility. G-17 is involved in the occurrence and development of gastric cancer and has a certain effect on the growth and deterioration of cancer cells. Consistent with previous research results, the G-17 level in CG was significantly increased. Besides, LPD significantly decreased the levels of proinflammatory factors in CG rats, including TNF-*α*, IL1B, and IL6, while remarkably improving anti-inflammatory factor levels, such as IFNG and IL10.

## 5. Conclusions

In this study, we explored the molecular mechanism of the therapeutic effect of LPD on CG from the perspective of NP, initially explained the theoretical connotation of LPD in the treatment of CG, and provided new ideas for the clinical treatment of CG. In addition, the construction of the “compound-target-disease” interaction network revealed that LPD may play a role in the treatment of CG through multiple components, multiple targets, and multiple pathways. This study provides a reference for the further molecular mechanism study of LPD in the treatment of CG. Of course, there were some limitations in the present study. First, the effective compounds and targets of action obtained in this study come from the currently clear database information, so our results only revealed the mechanism of LPD treatment of CG to a certain extent. Second, this study only validated the Jak-STAT signaling pathway, and we have not conducted in-depth studies on other biological processes that may be involved. Therefore, we need to search the relevant databases extensively to obtain comprehensive information about the compounds and targets of LPD. Furthermore, it is necessary to further conduct more in-depth studies of the possible pathways of LPD in the treatment of CG to clarify the mechanism of it in the treatment of CG.

## Figures and Tables

**Table 1:** The candidate compounds of LPD predicted by NP analyses.

Compound CID no.	Compounds	OB	DL	Score cutoff	Betweeness centrality	Closeness centrality	Degree	Precision targets	Attribution
CID: 5280343	Quercetin	46.43	0.28	—	0.3120	0.4757	16	1, 3, 5, 6, 7, 9, 10, 14, 18, 20, 25, 26, 27, 28, 30	CR
CID: 9294	Ephedrine	—	—	≥20	0.1770	0.4298	14	1, 2, 3, 4, 6, 7, 11, 12, 13, 14, 16, 20, 24, 27	PT
CID: 1549018	Jasmone	—	—	≥20	0.0891	0.3858	8	2, 5, 6, 14, 16, 17, 18, 23	CJ
CID: 5570	Trigonelline	—	—	≥20	0.0611	0.3858	8	1, 3, 5, 7, 13, 14, 15, 19	PT
CID: 5281232	Crocetin	35.30	0.26	—	0.0372	0.3740	7	1, 2, 13, 14, 15, 19	CJ
CID: 222284	*β*-Sitosterol	—	—	≥20	0.0192	0.3500	6	14, 18, 27	PT
CID: 325601	*β*-Asarone	—	—	≥20	0.0175	0.3630	4	10, 14, 22	AT
CID: 6287	Valine	—	—	≥20	0.0388	0.3798	4	1, 8, 14, 22	PT

**Table 2:** The information of common target proteins and their correlation score.

No.	Uniprot no.	Gene name	Protein name	Correlation score
1	P01584	IL1B	Interleukin-1 beta	62.57
2	P01375	TNF	Tumor necrosis factor	58.67
3	P05231	IL6	Interleukin-6	48.41
4	P18510	IL1RN	Interleukin-1 receptor antagonist protein	46
5	P04637	TP53	Cellular tumor antigen p53	45.65
6	P22301	IL10	Interleukin-10	43.33
7	P01579	IFNG	Interferon gamma	42.47
8	O00206	TLR4	Toll-like receptor 4	39.58
9	P10145	CXCL8	Interleukin-8	37.51
10	P29965	CD40LG	CD40 ligand	34.42
11	P25445	FAS	Tumor necrosis factor receptor superfamily member 6	30.39
12	P01589	IL2RA	Interleukin-2 receptor subunit alpha	30.07
13	P48023	FASLG	Tumor necrosis factor ligand superfamily member 6	27.17
14	P35354	PTGS2	Prostaglandin G/H synthase 2	26.04
15	P35225	IL13	Interleukin-13	25.29
16	P05112	IL4	Interleukin-4	24.55
17	Q9BZS1	FOXP3	Forkhead box protein P3	24.25
18	P01137	TGFB1	Transforming growth factor beta-1 proprotein	24.04
19	P05109	S100A8	Protein S100-A8	23.62
20	P60568	IL2	Interleukin-2	22.6
21	P19838	NFKB1	Nuclear factor NF-kappa-B p105 subunit	22.18
22	P35228	NOS2	Nitric oxide synthase, inducible	22.15
23	Q9UBU3	GHRL	Appetite-regulating hormone	21.36
24	P12821	ACE	Angiotensin-converting enzyme	21.36
25	P10451	SPP1	Osteopontin	20.73
26	P01133	EGF	Proepidermal growth factor	20.52
27	Q14790	CASP8	Caspase-8	20.44
28	P05164	MPO	Myeloperoxidase	20.19
29	P10645	CHGA	Chromogranin-A	20.07
30	P24385	CCND1	G1/S-specific cyclin D1	20

## Data Availability

All data used to support the findings of this study are available from the corresponding author upon request.

## Abbreviations

CG: Chronic gastritis
TCM: Traditional Chinese medicine
LPD: Lianpu Drink
KEGG: Kyoto Encyclopedia of Genes and Genomes
CR: Coptidis Rhizoma
MOR: *Magnolia officinalis Rehd Et Wils*.
CJ: *Cape jasmine*
PT: *Pinellia ternata*
AT: *Acorus tatarinowii*
FSB: Fermented soya beans
RR: Reed rhizome
NP: Network pharmacology
OB: Oral bioavailability
DL: Drug-like properties
PPI: Protein-protein interaction
SD: Sprague Dawley
MNNG: N-methyl-N′-nitro-N-ni-trosoguanidine
PG: Pepsinogen
G-17: Gastrin-17
PGR: Rate of PG I to PG II
c-Myc: Proto-oncogene.
